# Indirect Photodegradation of Sulfamethoxazole and Trimethoprim by Hydroxyl Radicals in Aquatic Environment: Mechanisms, Transformation Products and Eco-Toxicity Evaluation

**DOI:** 10.3390/ijms21176276

**Published:** 2020-08-30

**Authors:** Jiaoxue Yang, Guochun Lv, Chenxi Zhang, Zehua Wang, Xiaomin Sun

**Affiliations:** 1 Environment Research Institute, Shandong University, Qingdao 266237, China; yjx12stu@163.com (J.Y.); lgcttxs@foxmail.com (G.L.); sdzhw@mail.sdu.edu.cn (Z.W.); 2 College of Biological and Environmental Engineering, Binzhou University, Binzhou 256600, China; sdzhangcx@163.com

**Keywords:** sulfamethoxazole (SMX), trimethoprim (TMP), hydroxyl radical (•OH), transformation mechanisms, photoproducts, eco-toxicity

## Abstract

The bacteriostatic antibiotics, sulfamethoxazole (SMX) and trimethoprim (TMP), have frequently been found in wastewater and surface water, which raises the concerns about their ecotoxicological effects. The indirect photochemical transformation has been proven to be an efficient way to degrade SMX and TMP. In this study, the reaction mechanisms of the degradation by SMX and TMF by OH radicals were investigated by theoretical calculations. Corresponding rate constants were determined and the eco-toxicity of SMX and TMP and its degradations products were predicted using theoretical models. The results indicate that the most favorable pathways for the transformation of SMX and TMP are both •OH-addition reaction of benzene ring site with lowest Gibbs free energy barriers (6.86 and 6.21 kcal mol^−1^). It was found that the overall reaction rate constants of •OH-initial reaction of SMX and TMP are 1.28 × 10^8^ M^−1^ s^−1^ and 6.21 × 10^8^ M^−1^ s^−1^ at 298 K, respectively. When comparing the eco-toxicity of transformation products with parent SMX and TMP, it can be concluded that the acute and chronic toxicities of the degraded products are reduced, but some products remain harmful for organisms, especially for daphnid (toxic or very toxic level). This study can give greater insight into the degradation of SMX and TMP by •OH through theoretical calculations in aquatic environment.

## 1. Introduction

Pharmaceuticals and personal care products (PPCPs) as emerging organic micropollutants in the environment have been receiving increased attention due to their possible adverse effects on the aquatic organism and human health [1,2,3,4,5]. Sulfamethoxazole (SMX) and trimethoprim (TMP), as important antibiotics, have been widely used in the treatment of human and animal diseases and infections [6,7,8,9]. Excessive amounts of SMX and TMP are discharged into water for a wide range transmission to wastewater treatment plants [10], which cannot be effectively removed by conventional wastewater treatment technologies [11,12,13,14]. This has led to widespread transport of pharmaceutical contaminants in aquatic environment around the world [15].

SMX has regularly been detected in wastewater effluents and surface water with a concentration range of 100–2500 ng/L and 60–150 ng/L, even in drinking water at 12 ng/L [16,17,18]. Previous studies have reported that the residual concentration of TMP are 0.1–5 µg/L in wastewater effluents. Additionally, traces of TMP (2.2–10.9 ng/L and 0–19.8 ng/L) have been detected in surface and drinking water [19,20,21,22,23]. Based on the concentrations of SMX and TMP in the aquatic environment, it may induce the ecotoxicological concern about the chronic exposure of bacteria to trace level of antibiotics [24]. More importantly, the transformed products may retain the same level of biological properties as original compounds or even have a higher biological activity. Also, previous studies about the transformation of TMP exhibit some differences [25,26,27]. Thus, the chemical transformation of SMX and TMP deserve more attention due to their biological activity and adverse effects on aquatic ecosystems.

Much efforts have been spent on researching the degradation and environmental impact of SMX and TMP in aquatic environment. When compared with biodegradation, the photodegradation of SMX and TMP is identified as a predominant pathway in surface water [28,29,30]. Previous studies have focused on the degradation rate constant of SMX direct photolysis, which is 22.3 ± 1.86 h^−1^ at 3.2 pH [31]. In addition to the direct photolysis of pollutants, indirect photodegradation has been regarded as an important elimination way of organic contaminants, such as SMX and TMP in aquatic environment. In fact, advanced oxidation processes (AOPs) are broadly applied for removing organic materials of wastewaters [32,33]. Hydroxyl radical-based AOPs (such as UV/H_2_O_2_) treatments are strongly correlated with removal of pharmaceuticals with the rate constants on the order of 10^8^–10^9^ M^−1^ s^−1^ [34]. The experimental results shown that the rate constant for the •OH-initiated reaction of SMX is (5.8 ± 0.2) × 10^9^ M^−1^ s^−1^ [18,35]. In addition, many researchers have studied the degradation rate constants of SMX and TMP with carbonate radical (k = 2.68 × 10^8^ and 3.45 × 10^7^ M^−1^ s^−1^, respectively) [36]. When combined with the experimental research, a deeper understanding of the performance of SXM/TMP transformation mechanisms and kinetics is warranted.

Therefore, the objective of this work is to make a detailed elucidation for the indirect photochemical transformation and environmental impacts of SMX and TMP with •OH in aquatic environment by quantum chemical calculation and computational toxicology method. In this paper, the transformation mechanism and kinetic calculation of •OH-initiated reaction are expounded thoroughly. Furthermore, the ecotoxicity risk of SMX and TMP and their products during indirect photolysis process is evaluated while using the ECOSAR program. The study will provide theoretical insight into the indirect photochemical behavior and ecotoxicity prediction of SMX and TMP in aquatic environments.

## 2. Results and Discussion

### 2.1. Transformation Mechanisms of SMX and TMP with •OH

**Figure 1:**
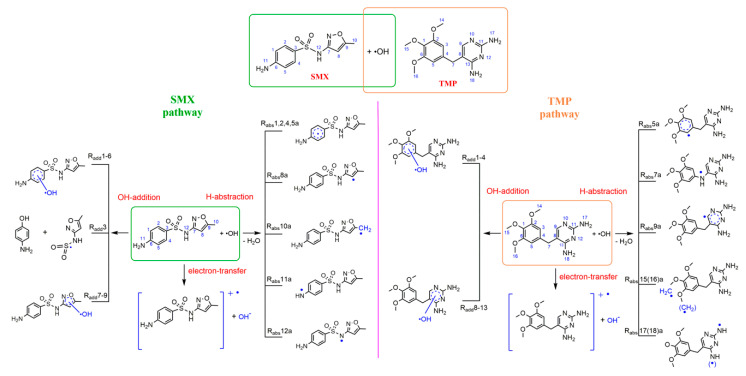
All the reaction pathways in the initial reaction of sulfamethoxazole (SMX) and trimethoprim (TMP) with •OH.

In this study, the indirect photochemical transformations of SMX and TMP towards •OH in aqueous phase were investigated. For the initial reaction of SMX and TMP with •OH, the degradation mechanisms are described in Figure 1. •OH attack SMX and TMP through the three basic pathways: (i) hydrogen abstraction (R_abs_), •OH extracts one H atom from SMX and TMP to form the dehydrogenated radical intermediates and H_2_O; (ii) hydroxylation reaction (R_add_), •OH adds to the C atom of reactants to form •OH-complexes; and, (iii) electron transfer (R_set_), the single-electron transfer from the reactants to •OH and generating the radical cation of reactants and •OH^−^.

#### 2.1.1. •OH-Initiated Reaction of SMX

For the R_set_ process of SMX, the radical cation species (SMX^+^•) and hydroxyl anion (OH^−^) were formed via the electron transfer. The reaction of SMX with •OH is an endothermic process by R_set_ pathway, which absorbs 26.33 kcal mol^−1^ of reaction energy. The results indicate that the R_set_ pathway is not a spontaneous reaction, and it is not easy to occur by single-electron transfer mechanism of SMX from a thermodynamic standpoint. In addition, the Marcus theory was used to calculate the energy barrier of the R_set_ pathway ($\Delta G_{SET}^{\neq }$= 33.08 kcal mol^−1^), further implying that the reactant has a low reactivity during this process, and R_set_ process has low reaction activity during •OH-initiated degradation of SMX. In general, R_set_ pathway is not the primary mechanism during the •OH-initiated indirect photodegradation process in the aquatic environment. Thus, the two mechanisms (H-abstraction reaction and •OH-addition reaction) were taken into consideration, primarily in the following discussion.

**Figure 2:**
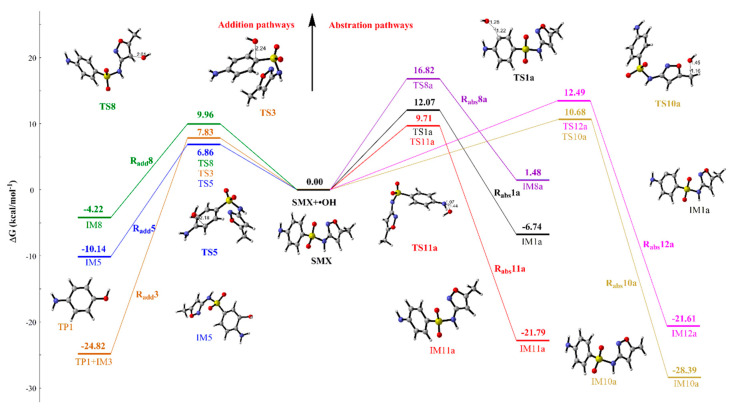
Schematic diagram of free energy for the reactions of SMX with •OH at different sites at the M06-2X/6–31+G (d, p) level.

•OH reacts easily with olefinic double bonds and anilines owing to the high reactivity. As shown in Figure 1, H atom of SMX can be divided to five categories (benzene ring, isoxazole, methyl, and two nitrogen-atoms), which provides the different reaction site for H-abstraction reaction (R_abs_). Additionally, hydroxylation reaction (R_add_) can occur at the benzene ring and isoxazole ring. The free energy profile for the •OH-initiated mechanism of the most favorable pathways of SMX and the optimized geometries of the major categories (namely, R_add_3, R_add_5, R_add_8, R_abs_1a, R_abs_8a, R_abs_10–12a) are provided in Figure 2. Figure S1 provides other pathways and Gibbs free energies. It is worth noting that the •OH-addition products are different at C3 site of benzene ring from other sites. The C-S bond breaks when •OH is added to C3 site, leading to the phenolic product. The Gibbs free energy barrier of the addition process (R_add_3) is 7.83 kcal mol^−1^. For the R_abs_ pathways, the free energy barriers are within a range of 9.71~16.82 kcal mol^−1^, which is higher than the R_add_ pathways (6.86~9.96 kcal mol^−1^). The results implying that, compared with H-abstraction reactions, hydroxylation pathways of SMX have higher reactivity in aquatic environments. In addition, the reaction energies of all •OH-initiated reaction are negative (−3.11~−28.39 kcal mol^−1^), except a small positive number (1.48 kcal mol^−1^) at C8 atom of abstraction reaction. The results mean that the R_add_ and R_abs_ pathways are exothermic processes, and they can be more favorable from the standpoint of thermodynamics. Comparing the thermodynamic and kinetic data of •OH-initiated reaction at different sites of SMX, the addition reaction occurred at C5 site on the benzene ring is the most advantageous channel for the degradation of SMX. The conclusion is verified by the lowest Gibbs free energy barrier (6.86 kcal mol^−1^) and the highest rate constants (6.18 × 10^7^ M^−1^ s^−1^) of R_add_5 pathway. In summary, R_add_ and R_abs_ pathways predictably play an important role in the •OH-initiated photodegradation mechanisms of SMX in an aquatic environment.

#### 2.1.2. •OH-Initiated Reaction of TMP

The indirect photolysis mechanisms of •OH-initiated reaction of TMP are similar with the photochemical transformations of SMX. For the R_set_ pathway, the single-electron transfer from the TMP to •OH. The radical cation intermediate (TMP^+^•) and hydroxyl anion (OH^−^) are formed in this process. The Gibbs free energy of activation ($\Delta G_{SET}^{\neq }$) of the SET reaction is 23.13 kcal mol^−1^ calculated by Marcus theory at 298.15 K. Simultaneously, the R_set_ mechanism of •OH-initiated reaction is endothermic with 21.88 kcal mol^−1^ of reaction energy. The results mean that the electron transfer reactions are not easy to occur from a thermodynamic point of view.

**Figure 3:**
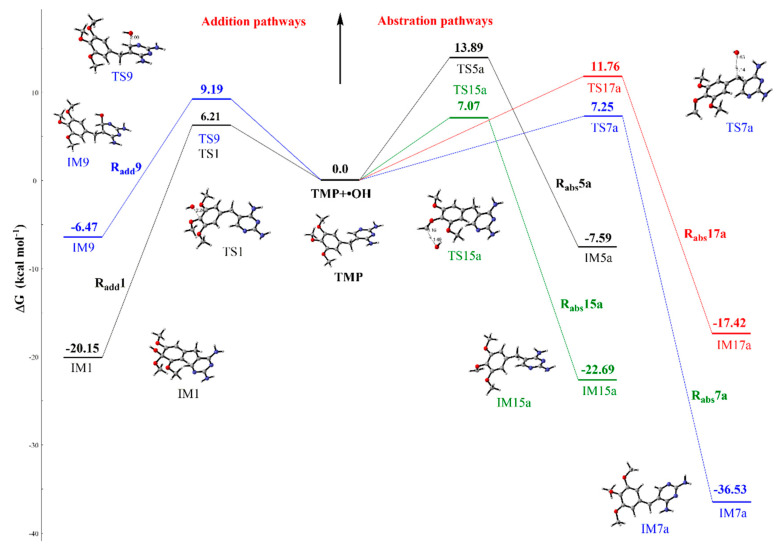
Schematic diagram of free energy for the reactions of TMP with •OH at different sites at the M06-2X/6–31+G (d, p) level.

Figure 1 also shows that the hydroxylation reaction (R_add_) and H-abstraction reaction (R_abs_) of TMP could occur at different sites (including benzene ring, five-membered heterocycle, and substituent group). Figure 3 clarifies the profile of the free energy for the •OH-initiated mechanisms of the most favorable pathways of the three major categories. Additionally, other channels of •OH-initiated reactions are described in Figure S2. The four pathways (R_add_1–4) of the benzene ring for the hydroxylation reactions are considered due to the symmetric structure of TMP. The lowest Gibbs free energy barrier of the addition reaction is 6.21 kcal mol^−1^ at C1 site, and the process releases up to 20.15 kcal mol^−1^ of reaction energy, as shown in Figure 3. For R_abs_ channels, the Gibbs free energy barrier of R_abs_15a (substituent group C15 site) is the lowest. The reaction needs to overcome the transition state (TS15a) with the energy barrier of 7.07 kcal mol^−1^ and release heart of 22.69 kcal mol^−1^. Furthermore, among the overall addition and abstraction pathways, the reaction energies that are released by •OH-initiated reaction range from −0.90 to −36.53 kcal mol^−1^. Meanwhile, the Gibbs free energy barriers of R_add_ and R_abs_ pathways are 6.21~18.42 kcal mol^−1^. All of the intermediates of •OH-initiated reactions of TMP have one free electron, which can lead to the occurrence of subsequent reaction.

When considering all of the •OH-initiated photolysis reaction of SMX and TMP, it is clear that hydroxylation reaction and H-abstraction mechanisms mainly contribute to the photochemical transformation of SMX and TMP in an aquatic environment. In particularly, the most favorable pathway of the transformation is hydroxylation reaction of benzene ring site.

### 2.2. Kinetic Calculation

The kinetic calculation is carried out using the TST method in order to further confirm the dominant pathways at the suitable temperature ranging of 273–328 K. The rate constants of the •OH-initiated degradation of SMX and TMP are calculated based on the thermodynamic data in aquatic environment. The bimolecular rate constants of the •OH-initiated reaction are calculated according to the TST method, as shown before. The initial reaction of •OH can be described as:

$$\mathrm{SMX}\;(\mathrm{TMP})+•\mathrm{OH}\;\overset{k_{1}}{\to }\;•\mathrm{OH}\;\mathrm{adducts}\;\mathrm{or}\;\mathrm{dehydrogenated}\;\mathrm{intermediates}$$

The total rate constant can be expressed using the formula:

*k_total_* = *k*_1_ (1)

The rate constants of •OH-addition and H-abstraction reaction are defined as *k*_add_ and *k*_abs_, respectively. The rate constants (*k*_add_, *k*_abs_, and *k*_total_) of the •OH-initiated reaction of SXM and TMP at different site are displayed in Tables S1 and S2, in detail. The overall rate constants (*k*_total_) at 273–328 K and experimental rate constants of SMX and TMP are given in Table 1. The calculated overall rate constant of SMX and TMP are 1.28 × 10^8^ M^−1^ s^−1^ and 6.21 × 10^8^ M^−1^ s^−1^ at 298 K, respectively. Additionally, the rate constants of bimolecular reaction of SMX and TMP with •OH are obtained by the experimental method [18,37]. The experimental results of SMX and TMP ((5.8 ± 0.2) × 10^9^ M^−1^ s^−1^ and 8.66 × 10^9^ M^−1^ s^−1^, respectively.) are an order of magnitude higher than the theoretical values, which may be due to the influence of concentration of reactants, PH, aqueous medium, catalyst, and some intermediates in the experiments. In addition, as the temperature increases, the total rate constants of SMX and TMP degradation decreases. For example, the total rate constant for TMP of the •OH-initiated reaction is 9.72 × 10^8^ M^−1^ s^−1^ at 273 K, which is 2.4 times higher than that at 328 K (4.03 × 10^8^ M^−1^ s^−1^). The highest rate constants of •OH-initiated reaction are *k*_add_5 (C5 site of SMX), *k*_add_1 (C1 site of TMP), which are corresponding with the thermodynamic results, as shown in Tables S1 and S2. Thus, the initiated reaction at C5 site of SMX (or at C1 site of TMP) is most favorable.

**Figure 4:**
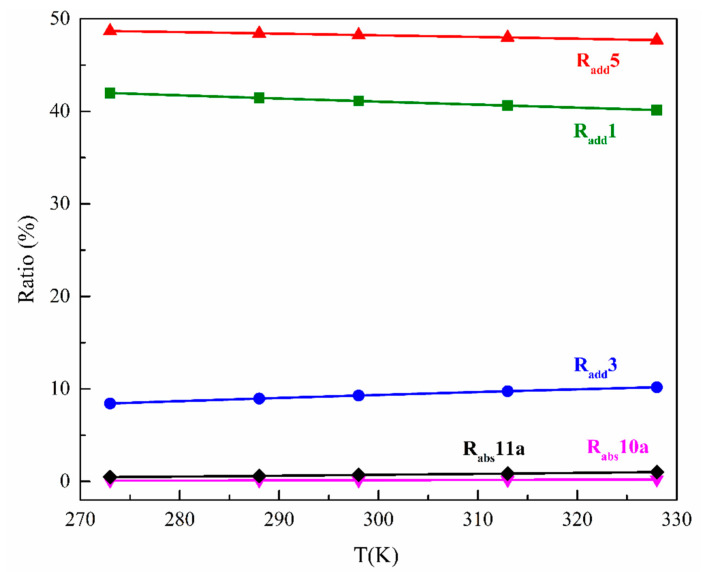
Calculated branching ratio of each route in the initial reaction of SMX with •OH at 273–328 K.

Furthermore, the contributions of initiated reactions at different site to the total reactions of SMX are expressed as a rate branching ratio, which can be expressed as *r_x_* =*k_X_*/*k_total_*. The calculated results *r_x_* are shown in Figure 4. The three paths (R_add_1, R_add_3, and R_add_5) of hydroxylation and two paths (R_abs_10a and R_abs_11a) of H-abstraction reactions that are most likely to occur are select to characterize the branching ratio of rate constants. It is obvious that the R_add_1 and R_add_5 pathways of •OH-addition reactions play a dominant role in the photochemical transformation. The rate branching ratio of these two pathways is determined as high as 89% at 298 K, and the ratio of R_add_3 (C3 site) is 9% at 298 K. In a previous study, Solar et al. [38] found that the reaction of •OH with the *ortho*-position (C1 or C5) of aniline is predominant (account for 54%), and 10% with *para*-position (C3) by the absorbance characteristics experiment. The results confirmed the rate branching ratio that was carried out by theoretical calculation. Thus, the hydroxylation reaction is significant to evaluate the subsequent transformation reaction of SMX.

### 2.3. Subsequent Transformation Reaction of SMX and TMP

**Figure 5:**
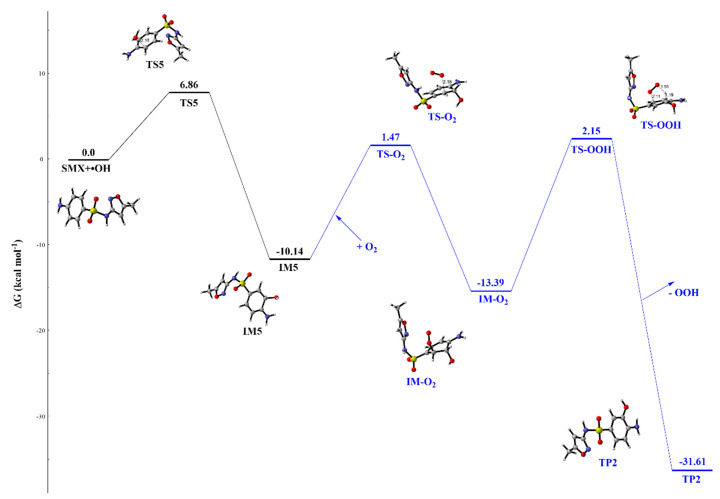
Schematic diagram of free energy for the subsequent reactions of IM5 during the transformation of SMX at the M06-2X/6–31 + G (d, p) level.

The intermediates generated from the •OH-initiated reaction of SXM and TMP contain a free radical, which can undergo the subsequent reactions due to the high reactivity. For the purpose of identifying the transformation products of SMX during the indirect photolysis process, the intermediate IM5 was selected to study the subsequent transformation, owing to the fastest reaction rate constant. Figure 5 shows the profile of free energy for subsequent reaction of IM5. The subsequent reactions of intermediate IM5 are considered in the presence of O_2_ in aquatic environment, as shown in Figure 5. The reaction of IM5 with O_2_ is exothermic, which releases the reaction energy of 3.25 kcal mol^−1^. In the process, the Gibbs free energy barrier of transition state TS-O_2_ is 11.61 kcal mol^−1^. Subsequently, the intermediate IM-O_2_ can undergo the hydrogen peroxide radical stripping reaction with the Gibbs free energy barrier of 15.54 kcal mol^−1^, leading to the formation of SMX transformation product (TP2). Simultaneously, the process can release the reaction energy of 28.36 kcal mol^−1^. The product was identified by the triple quadrupole TOF mass spectrometer in the experiment [35].

**Figure 6:**
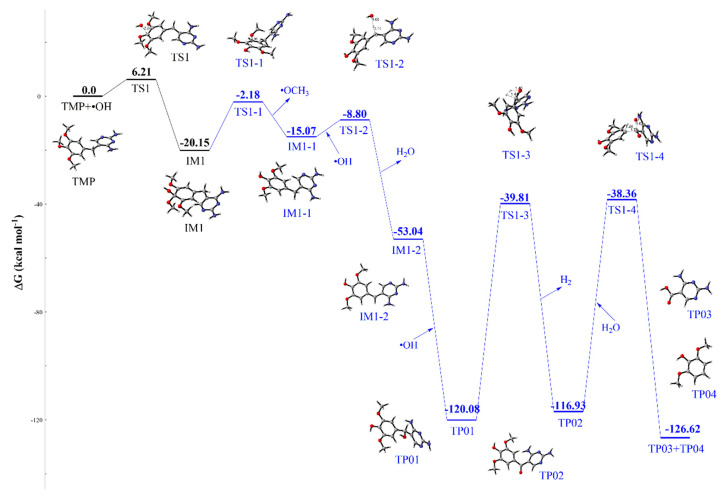
Schematic diagram of free energy for the subsequent reactions of IM1 during the transformation of TMP at the M06-2X/6–31+G (d, p) level.

Furthermore, the intermediate IM1 of TMP is considered in the photodegradation process. Figure 6 depicts the transformation pathways of IM1 with blue color. IM1 can undergo demethylation reaction to form the IM1-1 and •OCH_3_ radical. The reaction requires the free energy barrier of 17.97 kcal mol^−1^, and the channel is endothermic with a little reaction energy of 5.08 kcal mol^−1^. Subsequently, the product TP01 is formed through hydroxylation reaction at C7 site. The formation process includes two steps, corresponding to the reactions IM1-1 + •OH → IM1-2, and IM1-2 → TP01. In this process, the intermediate IM1-2 is produced through the transition state TS1-2 with the Gibbs free energy barrier of 6.27 kcal mol^−1^. In addition, the next addition reaction of IM1-2 with •OH is barrier-less process and a lot of reaction energy of 67.04 kcal mol^−1^ is released at the same time. The ketone product TP02 is generated through oxidation reaction by losing of a molecular H_2_. In this process, the oxidizing agent captures the H from the carbon of C7 site and •OH, and it forms the C = O bond. The reaction is not easy to occur with a high Gibbs free energy barrier of 80.27 kcal mol^−1^ in theory. However, in natural water environment, the reactivity may be enhanced by the catalytic action of oxidant. Next, TP02 reacts with H_2_O via the hydrolysis reaction (Gibbs free energy barriers of 78.57 kcal mol^−1^), into TP03 (2,4-diaminopyrimidine-5-carboxylic acid, DAPC), and TP04 simultaneously with releasing energy of 6.69 kcal mol^−1^. The transformation products (TP01, TP02 and TP03) of TMP were verified by HRMS and MS^2^ method in experiment [39].

The mechanism studies elucidate that •OH can attack the organic contaminant (SMX and TMP) and the two intermediates (IM5 of SMX and IM1 of TMP) are easily formed through the •OH-addition pathway, according to above discussion. Furthermore, the transformed products of SMX (TP1 and TP2), also with the photolysis products of TMP (TP 01, TP02, TP03, and TP04), are formed in the indirect photochemical transformation process. The ecological risks of these products need to be further considered in the aquatic environment.

### 2.4. Eco-Toxocity Evaluation

In this work, QSAR analysis calculated by ECOSAR program was applied to predict the aquatic toxicity for fish, daphnid, and green algae, including acute toxicity and chronic toxicity. In addition, the values of 96-h median lethal concentration (LC_50_) for fish, 48-h LC_50_ for daphnid, and 96-h median effective concentration (EC_50_) for green algae were used to analysis the acute toxicity. Additionally ChV is defined as the geometric mean of the unobserved effect concentration and the lowest observed effect concentration. Based on the results of EC_50_ for D. *magna* and LC50 for O. *latipes*, the values of aniline derivative (unhindered) are selected for preliminary prediction of SMX, TMP, and transformation products [40,41].

**Figure 7:**
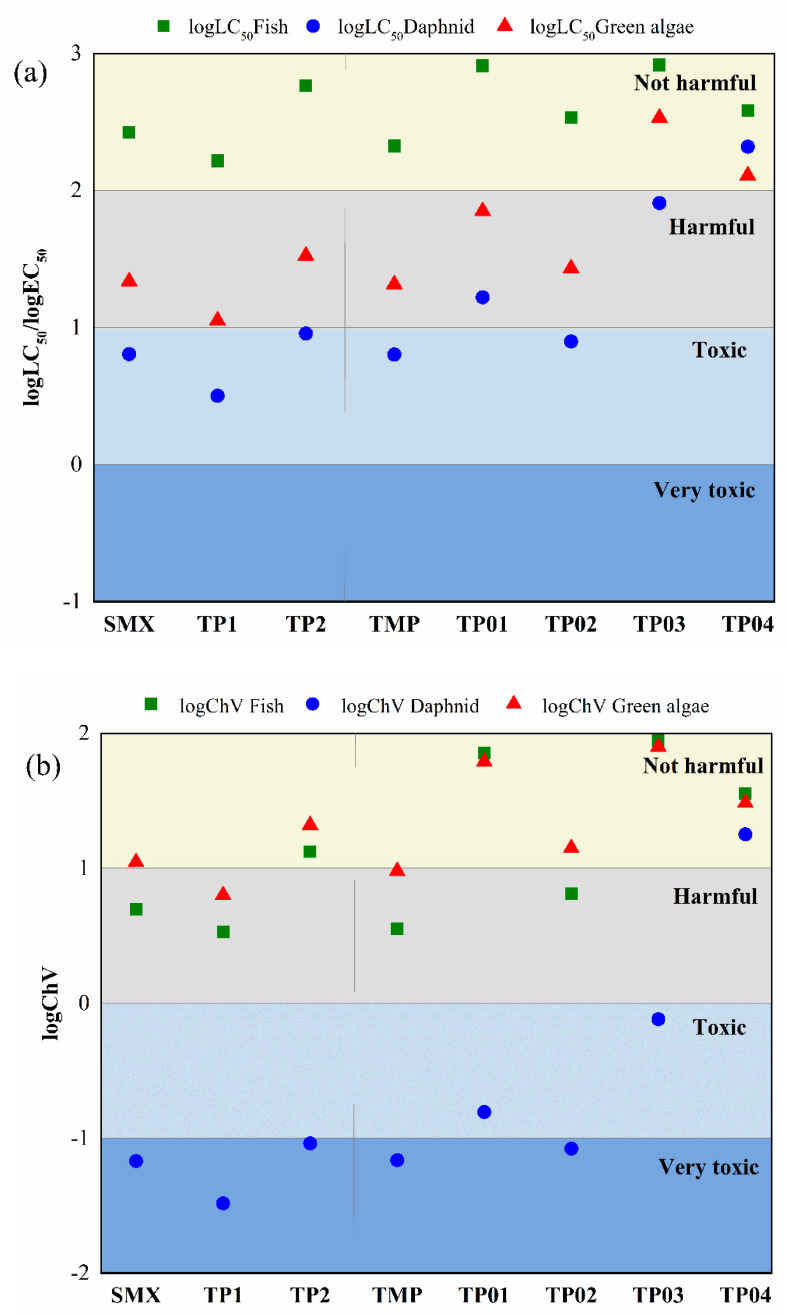
Acute toxicity (**a**) and chronic toxicity (**b**) (unit: mg L^−1^) of SMX and TMP and their transformation products.

Figure 7 illustrates the acute and chronic toxicities of SMX, TMP, and transformation products. Obviously, the acute and chronic toxicity of SMX, TP1, and TP2 to the three aquatic organisms express the same trend. The chronic toxicity of SMX and TPs for daphnid is very low, less than 0.1 mg L^−1^, which classified as very toxic substances according to Chinese hazard evaluation guidelines (Table S3). Additionally the product TP2 shows lower toxicity than SMX, which may be due to the increased hydrophilicity of the hydroxylated product of SMX.

The aquatic toxicity of most transformation products for fish and green alage are lower than TMP, as shown in Figure 7. The results indicate that the •OH-initiated degradation process of TMP reduced the toxicity of products. Although the acute toxicity decreases with the transformation of TMP, some of the products are classified as toxic and harmful to daphnid green alage. Similar with SMX, the daphnid is the most sensitive organism for aquatic toxicity. In summary, the eco-toxicity assessment of transformation products and parent compounds (SMX and TMP) become a necessary consideration during the degradation process of contaminants in aquatic environment.

## 3. Materials and Method

### 3.1. Mechanism Calculation

In this study, all of the quantum chemical calculations are implemented using the Gaussian 09 program [42]. The geometries of reactants, intermediates, transition states (TS), and products, as well as its vibrational frequencies are optimized with M06-2X functionals at a level 6-31 + G (d,p) basis set [43]. The M06-2X method, as a density functional theory (DFT) model, has been successfully applied to the mechanism studies of organic contaminants in the aquatic environment. [44,45,46,47,48,49]. The purpose of vibrational frequency calculation is to confirm the structure of transition states and local minimal point. Meanwhile, intrinsic reaction coordinate (IRC) calculations [49] are analyzed in order to verify the transition states that are connected the reactants with their corresponding products for the transformation pathway. Moreover, for a more accurate evaluation of energetic parameters, the single point energies of all of the geometries were carried out at the M06-2X/6-311++G (3df,3pd) level. Furthermore, the influence of solvent water is evaluated using the polarized continuum model (PCM) within a self-consistent reaction field (SCRF) theory [50,51,52]. The PCM has been proven to be flexible and accurate, in particularly, when the solute is accommodated in a cavity of realistic molecular shape and it has been widely used for the study of many chemical processes [53].

In addition, the Marcus theory [54] was used to calculate the mechanism of single-electron transfers (SET) pathway. The SET activation barrier ($\Delta G_{SET}^{\neq }$) is defined relying on the reaction free energy ($\Delta G_{SET}$) and the nuclear reorganization energy (λ):

(2) $$\Delta G_{SET}^{\neq }=\frac{{\left(\lambda +\Delta G_{SET}\right)}^{2}}{4\lambda }$$

The reorganization energy (λ) is determined by two energies, which has been calculated as:

(3) $$\lambda =\Delta E_{SET}-\Delta G_{SET}$$

where $\Delta E_{SET}$ is the energy difference between reactants and vertical products, which changes the spin multiplicity and charge at the same geometries.

### 3.2. Kinetics Computation

The transition state theory (TST) has been used to calculate the reaction kinetics, which has been successfully used to deal with radical reaction in former studies [55,56,57]. In this work, the rate constants of elementary reactions have been carried out with the effective method according to the activation energy barrier over a suitable temperature range. The rate constants are calculated by KiSThelP package that is based on TST with Wigner tunneling correction [58]. In order to obtain the rate constants of aqueous bimolecular reaction, the calculated rate constants need to be converted into corresponding aqueously values by dividing a coefficient (k_c_), k_c_ is expressed by the following formular:

k_c_ = RT/P^θ^ (4)

where R, T, and P^θ^ are gas constant, temperature, and standard atmospheric pressure, respectively.

### 3.3. Ecotoxicity Evaluation

The ecological structure-activity relationships (ECOSAR) model, as a practical method for calculating toxicity, is employed to predict the ecotoxicity of SMX, TMP, and their transformation products [59,60]. The program can be used to estimate toxicity at the screening level of organic contaminants [61]. The eco-toxicity is evaluated by the acute toxicity (LC_50_ and EC_50_) and chronic toxicity (ChV) of SMX, TMP, and their transformation products for three aquatic organisms (green algae, daphnia, and fish).

## 4. Conclusions

In this paper, the transformation mechanisms, rate constants, and ecological risks for the •OH-initiated degradation process of SMX and TMP have been studied by quantum chemistry and computational toxicology methods. The lowest Gibbs free energy barrier of SMX (or TMP) initiated by •OH is 6.86 (or 6.21) kcal mol^−1^ through the R_add_5 (or R_add_1) pathway, which indicate that the most favorable pathways of the transformation are both •OH-addition reaction of benzene ring site. It is worth noting that the results shown that the hydroxylation of SMX at C3 site leading to the breaking the C-S bond and the opening of aromatic ring. This is an important finding that •OH radicals alone are able to cause the opening of aromatic ring. In addition, the rate constants of SMX and TMP were calculated for the assessment of the aquatic environmental fate. The overall rate constants of SMX and TMP reacted with •OH were 1.28 × 10^8^ M^−1^ s^−1^ and 6.21 × 10^8^ M^−1^ s^−1^ at 298 K, respectively. Additionally, the R_add_1 and R_add_5 pathways (*ortho*-position of aniline) of hydroxylation reactions of SMX play a dominant role (as high as 89% of branching ratio) in the photochemical transformation. Moreover, the hydroxylation products are formed through the subsequent reaction of •OH-complexes of SMX and TMP. It is noteworthy that although the aquatic toxicity decreases with the degradation of SMX and TMP, many products still maintain harmful levels to organisms, especially to daphnid (toxic or very toxic level).

## Figures and Tables

**Table 1:** Calculated overall rate constants (M^−1^ s^−1^) between 273 and 328 K in the reaction of SMX and TMP with •OH.

**T(K)**	**SMX**	**Experiment**	**TMP**	**Experiment**
273	1.58 × 10^8^	(5.8 ± 0.2) × 10^9^	9.72 × 10^8^	8.66 × 10^9^
288	1.39 × 10^8^		7.35 × 10^8^	
298	1.28 × 10^8^		6.21 × 10^8^	
313	1.15 × 10^8^		4.94 × 10^8^	
328	1.05 × 10^8^		4.03 × 10^8^	

## Supplementary Materials

Supplementary materials can be found at https://www.mdpi.com/1422-0067/21/17/6276/s1. Figure. S1: The pathways of •OH-initiated reaction of SMX in aquatic environment with the Gibbs free energy barriers ΔE_b_ and reaction energies ΔE_r_ (unit: kcal mol^−1^). Figure. S2: The pathways of •OH-initiated reaction of TMP in aquatic environment with the Gibbs free energy barriers ΔE_b_ and reaction energies ΔE_r_ (unit: kcal mol^−1^). Table S1: Calculated rate constants (M^−1^ s^−1^) between 273 and 328 K in the reaction of SMX and •OH. Table S2: Calculated rate constants (M^−1^ s^−1^) between 273 and 328 K in the reaction of TMP and •OH. Table S3: The acute and chronic toxic criterion (unit: mg L^−1^).
