# Supplementary material for: Indirect Photodegradation of Sulfamethoxazole and Trimethoprim by Hydroxyl Radicals in Aquatic Environment: Mechanisms, Transformation Products and Eco-Toxicity Evaluation
Source: Int J Mol Sci. 2020 Aug 30;21(17):6276. doi: 10.3390/ijms21176276 (PMC7503993; doi:10.3390/ijms21176276)
Supplement: Supplementary file 1 [file ijms-21-06276-s001.pdf]

Supplementary material for

Indirect photodegradation of sulfamethoxazole and trimethoprim by hydroxyl radical in aquatic environment: mechanisms, transformation products and eco-toxicity evaluation

Jiaoxue Yang[1], Guochun Lv[1], Chenxi Zhang[2], Zehua Wang[1], Xiaomin Sun[1, *]

[1] Environment Research Institute, Shandong University, Qingdao 266237, China

[2] College of Biological and Environmental Engineering, Binzhou University, Binzhou 256600, China

[*]Corresponding author: Xiaomin Sun, sxmwch@sdu.edu.cn

Figures and Table Captions

Figure. S1. The pathways of •OH-initiated reaction of SMX in aquatic environment with the Gibbs free energy barriers ΔE_b and reaction energies ΔE_r (unit: kcal mol^{-1})

Figure. S2. The pathways of •OH-initiated reaction of TMP in aquatic environment with the Gibbs free energy barriers ΔE_b and reaction energies ΔE_r (unit: kcal mol^{-1})

Table S1 Calculated rate constants (M^{-1} s^{-1}) between 273 and 328 K in the reaction of SMX and •OH

Table S2 Calculated rate constants (M^{-1} s^{-1}) between 273 and 328 K in the reaction of TMP and •OH

Table S3 The acute and chronic toxic criterion (unit: mg L^{-1})

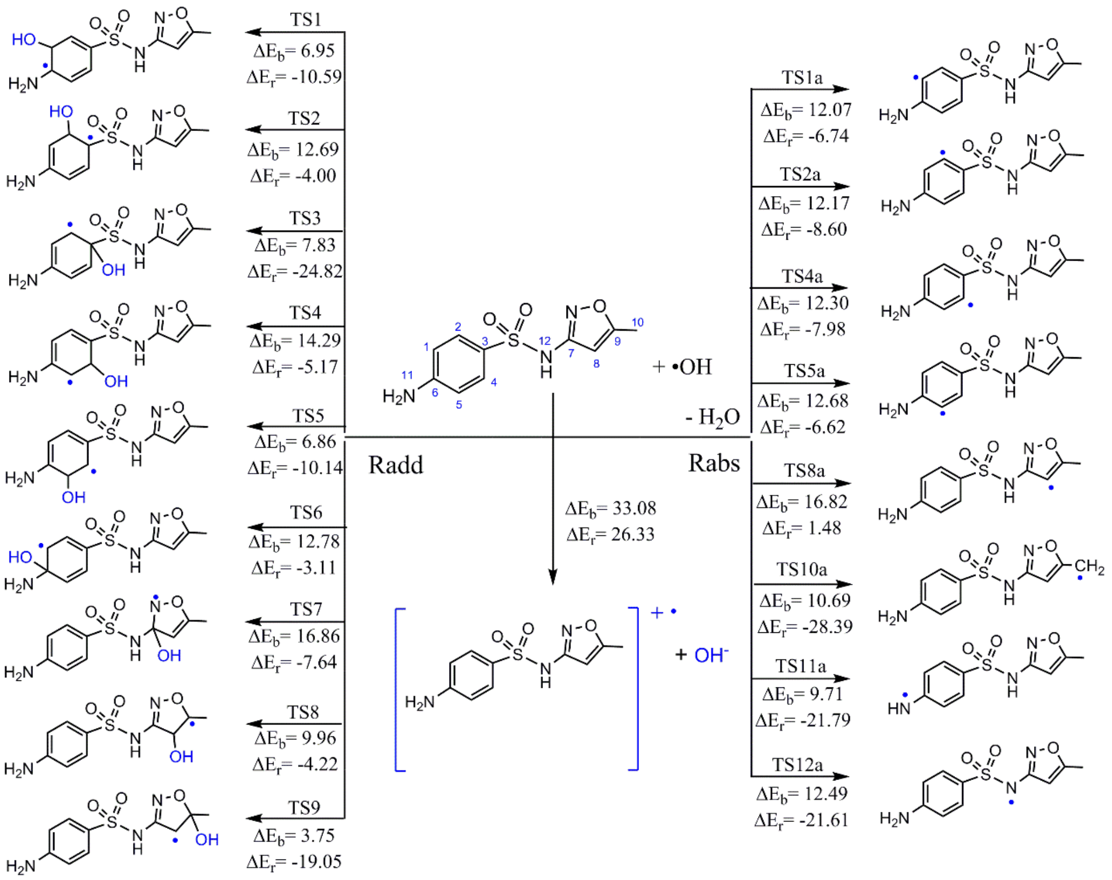

Figure. S1. The pathways of •OH-initiated reaction of SMX in aquatic environment with the Gibbs free energy barriers ΔE_b and reaction energies ΔE_r (unit: kcal mol^{-1})

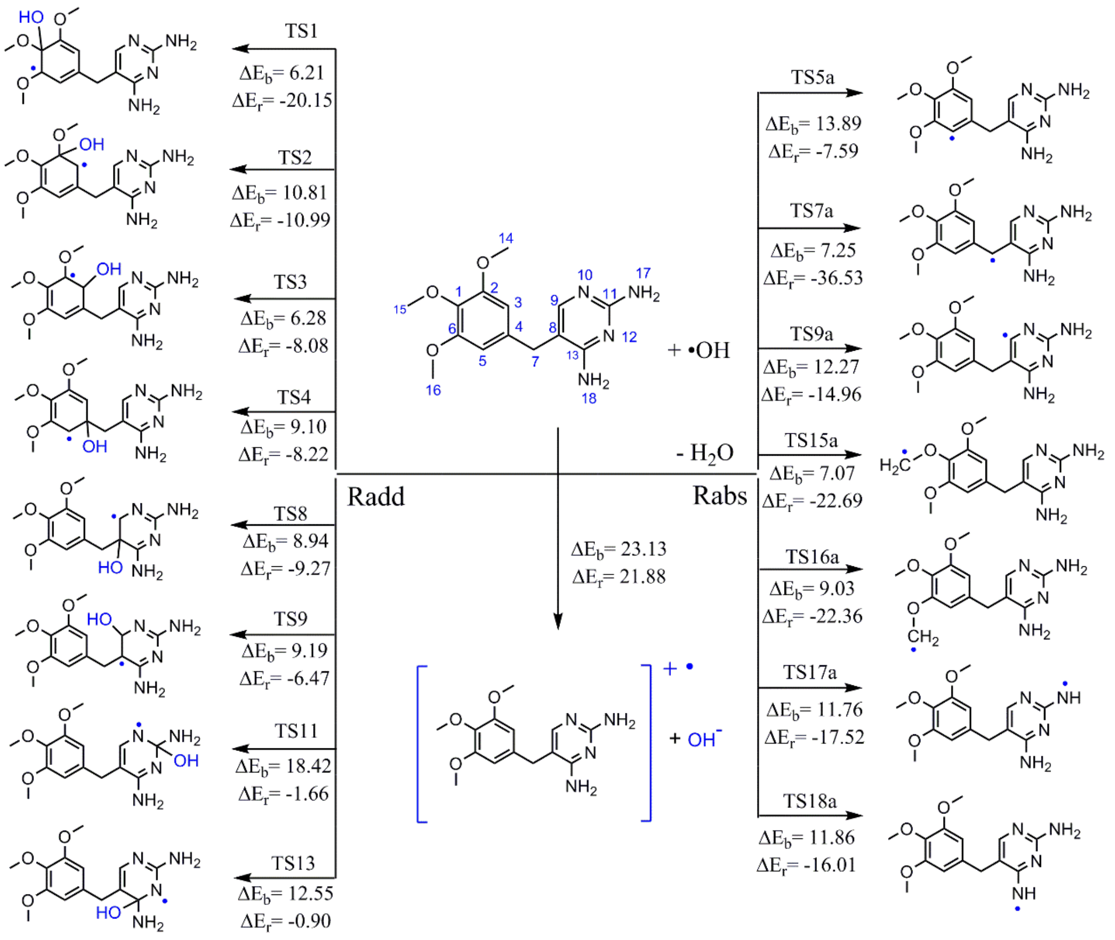

Figure. S2. The pathways of •OH-initiated reaction of TMP in aquatic environment with the Gibbs free energy barriers ΔE_b and reaction energies ΔE_r (unit: kcal mol^{-1})

Table S1 Calculated rate constants (M^{-1} s^{-1}) between 273 and 328 K in the reaction of SMX and •OH

T(K)	273	288	298	313	328
$R_{add}1$	6.65×10^7	5.75×10^7	5.27×10^7	4.68×10^7	4.21×10^7
$R_{add}2$	2.07×10^3	3.08×10^3	3.94×10^3	5.54×10^3	7.56×10^3
$R_{add}3$	1.34×10^7	1.24×10^7	1.19×10^7	1.12×10^7	1.07×10^7
$R_{add}4$	1.32×10^2	2.05×10^2	2.68×10^2	3.91×10^2	5.51×10^2
$R_{add}5$	7.71×10^7	6.72×10^7	6.18×10^7	5.53×10^7	5.00×10^7
$R_{add}6$	1.82×10^3	2.59×10^3	3.22×10^3	4.35×10^3	5.73×10^3
$R_{add}7$	1.47	2.64	3.78	6.19	9.73
$R_{add}8$	3.04×10^5	3.26×10^5	3.40×10^5	3.62×10^5	3.84×10^5
$R_{add}9$	1.73×10^5	1.85×10^5	1.93×10^5	2.05×10^5	2.17×10^5
$R_{abs}1a$	1.30×10^4	1.87×10^4	2.34×10^4	3.19×10^4	4.24×10^4
$R_{abs}2a$	2.09×10^4	2.42×10^4	2.66×10^4	3.02×10^4	3.40×10^4
$R_{abs}4a$	1.22×10^4	1.57×10^4	1.83×10^4	2.28×10^4	2.77×10^4
$R_{abs}5a$	4.64×10^3	6.74×10^3	8.47×10^3	1.16×10^4	1.56×10^4
$R_{abs}8a$	2.29	5.40	9.11	18.8	36.4
$R_{abs}10a$	1.35×10^5	1.61×10^5	1.79×10^5	2.09×10^5	2.40×10^5
$R_{abs}11a$	7.59×10^5	8.43×10^5	8.99×10^5	9.85×10^5	1.07×10^6
$R_{abs}12a$	2.05×10^4	2.54×10^4	2.89×10^4	3.45×10^4	4.06×10^4
k_{total}	1.58×10^8	1.39×10^8	1.28×10^8	1.15×10^8	1.05×10^8
experiment			$(5.8\pm0.2)\times10^9$		

Table S2 Calculated rate constants (M^{-1} s^{-1}) between 273 and 328 K in the reaction of TMP and •OH

T(K)	273	288	298	313	328
$R_{add}1$	2.89×10^{8}	2.17×10^{8}	1.82×10^{8}	1.43×10^{8}	1.16×10^{8}
$R_{add}2$	7.16×10^{4}	7.90×10^{4}	8.41×10^{4}	9.18×10^{4}	9.98×10^{4}
$R_{add}3$	2.58×10^{8}	1.91×10^{8}	1.59×10^{8}	1.23×10^{8}	9.82×10^{7}
$R_{add}4$	1.39×10^{6}	1.43×10^{6}	1.46×10^{6}	1.51×10^{6}	1.55×10^{6}
$R_{add}8$	2.91×10^{6}	2.74×10^{6}	2.40×10^{6}	1.95×10^{6}	1.77×10^{6}
$R_{add}9$	1.32×10^{6}	1.31×10^{6}	1.30×10^{6}	1.29×10^{6}	1.29×10^{6}
$R_{add}11$	0.07	0.17	0.29	0.61	1.18
$R_{add}13$	3.03×10^{3}	3.92×10^{3}	4.59×10^{3}	5.72×10^{3}	6.99×10^{3}
$R_{abs}5a$	6.19×10^{2}	9.35×10^{2}	1.20×10^{3}	1.71×10^{3}	2.36×10^{3}
$R_{abs}7a$	4.89×10^{7}	4.19×10^{7}	3.81×10^{7}	3.36×10^{7}	3.00×10^{7}
$R_{abs}9a$	2.71×10^{3}	2.63×10^{3}	2.50×10^{3}	2.39×10^{3}	2.27×10^{3}
$R_{abs}15a$	1.12×10^{8}	8.99×10^{7}	7.86×10^{7}	6.53×10^{7}	5.53×10^{7}
$R_{abs}16a$	2.20×10^{6}	2.32×10^{6}	2.40×10^{6}	2.52×10^{6}	2.65×10^{6}
$R_{abs}17a$	2.99×10^{4}	3.65×10^{4}	4.12×10^{4}	4.89×10^{4}	5.70×10^{4}
$R_{abs}18a$	4.02×10^{4}	4.91×10^{4}	5.54×10^{4}	6.53×10^{4}	7.59×10^{4}
k_{total}	9.72×10^{8}	7.35×10^{8}	6.21×10^{8}	4.94×10^{8}	4.03×10^{8}
experiment			8.66×10^{9}		

Table S3 The acute and chronic toxic criterion (unit: mg L^{-1})

Acute toxicity [1]	Chronic toxicity [2]	Toxicity grade
$LC_{50}>100$ or $EC_{50}>100$	$ChV>10$	Not harmful
$10< LC_{50}<100$ or $10< EC_{50}<100$	$1<ChV<10$	Harmful
$1< LC_{50}<10$ or $1< EC_{50}<10$	$0.1<ChV<1$	Toxic
$LC_{50}<1$ or $EC_{50}<1$	$ChV<0.1$	Very toxic

[1] Criteria set by the European Union (described in Annex VI of Directive 67/548/EEC)

[2] Criteria set by the Chinese hazard evaluation guidelines for new chemical substances (HJ/T 154–2004)
